# Editorial: Police education and training revisited: Drawbacks and advances

**DOI:** 10.3389/fpsyg.2022.1045924

**Published:** 2022-10-14

**Authors:** Mario S. Staller, Swen Koerner, Craig Bennell, Joel Suss

**Affiliations:** ^1^Department of Police, University of Applied Sciences of Police and Public Administration North Rhine-Westphalia, Aachen, Germany; ^2^Department of Martial Research and Training Pedagogy, German Sport University, Cologne, Germany; ^3^Department of Psychology, Carleton University, Ottawa, ON, Canada; ^4^Department of Psychology, Wichita State University, Wichita, KS, United States

**Keywords:** police learning, police–citizen interaction, reflexivity, scientific observation, professionalization and professional development

The education and training of police officers plays a prominent role in equipping officers with the knowledge structure, competencies, attitudes, and values that are needed to professionally conduct their duties in alignment with the ideals of a democratic society. Police officers learn in formal learning settings such as classrooms and scenario-based training rooms, but learning extends to a variety of non-formal and informal learning settings that exist outside the explicit curriculum of police education and training institutions (see “(Non-)Learning to police”). The “police system,” with all its structures and frameworks, as well as its individuals (e.g., police trainers, management, and supporting staff), shares the power—and the responsibility—for ensuring that what is learned is what is needed. However, current debates about police professionalization and reform, sparked in part by the death of George Floyd (Boxer et al., 2021), indicate that there is much to learn by focusing scientific scrutiny on police education and training. Observing and re-evaluating learning settings and goals through a scientific lens addresses one leverage point of the complex system that has repeatedly led to unfavorable outcomes in police–citizen encounters.

Although, organizationally, the “science system” is not necessarily part of the “police system,” the two social systems have become aligned over the last two decades, as evidenced by the rise of the term “evidence-based policing” (Boulton et al., 2020). Also, current debates concerning police–citizen encounters can be interpreted as progress in the sense that there *is* amplified communication about policing (Nassehi, 2021) and a growing acceptance that a scientific approach might benefit our understanding of police–citizen encounters. Understanding the factors that influence these interactions includes examining the education and training officers receive.

As researchers, we are fully aware that our logic related to the system of science fundamentally differs from the logic of the policing system. As such, we must regularly reflect on our share of, and contribution to, the status quo of police training and education. This is evidenced by discussions we have between ourselves (Bennell et al., 2021; Koerner and Staller, 2022). Yet, as researchers, we have the tools available that allow for rigorous and alternative observations (and the observation of our observations) that might provide a reflexive lens through which we can better understand issues around police education and training.

With the limitations of our own perspectives in mind, it is with delight that we share our Research Topic “*Police education and training revisited: Drawbacks and advances*” with the scientific community and with those we want to help and encourage in their practice: police trainers and police managers. With 10 original research reports,one brief report, two conceptual analyses, and one review, we provide a Research Topic—comprising 14 articles from a total of 50 dedicated authors—that sheds light on issues of performance in police–citizen encounters as well as on issues of the corresponding education and training that officers receive. By providing this Research Topic, we hope to foster further research concerning the Research Topic of police education and training, and stimulate discussion between practitioners, practitioners and researchers, and among researchers.

## Author contributions

MS and SK drafted the manuscript. CB and JS provided conceptual and Editorial contributions. All authors contributed to manuscript revision, read, and approved the submitted version.

## Conflict of interest

The authors declare that the research was conducted in the absence of any commercial or financial relationships that could be construed as a potential conflict of interest.

## Publisher's note

All claims expressed in this article are solely those of the authors and do not necessarily represent those of their affiliated organizations, or those of the publisher, the editors and the reviewers. Any product that may be evaluated in this article, or claim that may be made by its manufacturer, is not guaranteed or endorsed by the publisher.
